# RETRACTED: Wu et al. EGF-Enhanced GnRH-II Regulation in Decidual Stromal Cell Motility Through Twist and N-Cadherin Signaling. *Int. J. Mol. Sci.* 2023, *24*, 15271

**DOI:** 10.3390/ijms27219644

**Published:** 2026-10-28

**Authors:** Hsien-Ming Wu, Liang-Hsuan Chen, Hong-Yuan Huang, Hsin-Shih Wang, Chia-Lung Tsai

**Affiliations:** Department of Obstetrics and Gynecology, Chang Gung Memorial Hospital Linkou Medical Center, School of Medicine, Chang Gung University, Taoyuan 333, Taiwan; elsachen0119@gmail.com (L.-H.C.); hongyuan@cgmh.org.tw (H.-Y.H.); hswang0707@gmail.com (H.-S.W.); cltsai@cgmh.org.tw (C.-L.T.)

The journal retracts the article titled “EGF-Enhanced GnRH-II Regulation in Decidual Stromal Cell Motility through Twist and N-Cadherin Signaling” [1], cited above.

Following publication, concerns were brought to the attention of the Editorial Office regarding the integrity of figures presented in this publication.

In accordance with the journal’s standard procedures, the Editorial Office and Editorial Board conducted an investigation that identified indications of image duplication across Figures 1, 4 and 8 presented in [1], as well as multiple instances of panel duplication between Figure 1 of this article and Figures 3 and 5 of an earlier publication [2] by a similar group of authors. Although the authors cooperated during the investigation, the Editorial Board concluded that the concerns identified could not be satisfactorily resolved based on the information and materials provided.

As a result, the Editorial Board has lost confidence in the reliability of the findings reported in this article and has decided to retract the publication as per MDPI’s retraction policy.

This retraction was approved by the Editor-in-Chief of the *International Journal of Molecular Sciences*.

The authors have been informed of this retraction. The author Hsien-Ming Wu agreed to this decision. The remaining authors did not provide a comment on this decision.
